# ICTV Virus Taxonomy Profile: *Polyomaviridae*

**DOI:** 10.1099/jgv.0.000839

**Published:** 2017-06-22

**Authors:** Ugo Moens, Sébastien Calvignac-Spencer, Chris Lauber, Torbjörn Ramqvist, Mariet C. W. Feltkamp, Matthew D. Daugherty, Ernst J. Verschoor, Bernhard Ehlers, ICTV Report Consortium

**Affiliations:** ^1^​ University of Tromsø, 9037 Tromsø, Norway; ^2^​ Robert Koch Institute, 13353 Berlin, Germany; ^3^​ Technische Universität Dresden, Dresden, Germany; ^4^​ Karolinska Institutet, Stockholm, Sweden; ^5^​ Leiden University Medical Center, Leiden, the Netherlands; ^6^​ University of California, San Diego, USA; ^7^​ Biomedical Primate Research Centre, Rijswijk, the Netherlands

**Keywords:** *Polyomaviridae*, taxonomy, ICTV report, simian virus 40, budgerigar fledgling disease polyomavirus, Merkel cell polyomavirus, BK polyomavirus, JC polyomavirus

## Abstract

The *Polyomaviridae* is a family of small, non-enveloped viruses with circular dsDNA genomes of approximately 5 kbp. The family includes four genera whose members have restricted host range, infecting mammals and birds. Polyomavirus genomes have also been detected recently in fish. Merkel cell polyomavirus and raccoon polyomavirus are associated with cancer in their host; other members are human and veterinary pathogens. Clinical manifestations are obvious in immunocompromised patients but not in healthy individuals. This is a summary of the International Committee on Taxonomy of Viruses (ICTV) Report on the taxonomy of the *Polyomaviridae,* which is available at www.ictv.global/report/polyomaviridae.

## Abbreviation

LTAg, large T-antigen.

## Virion

**Fig. 1.**
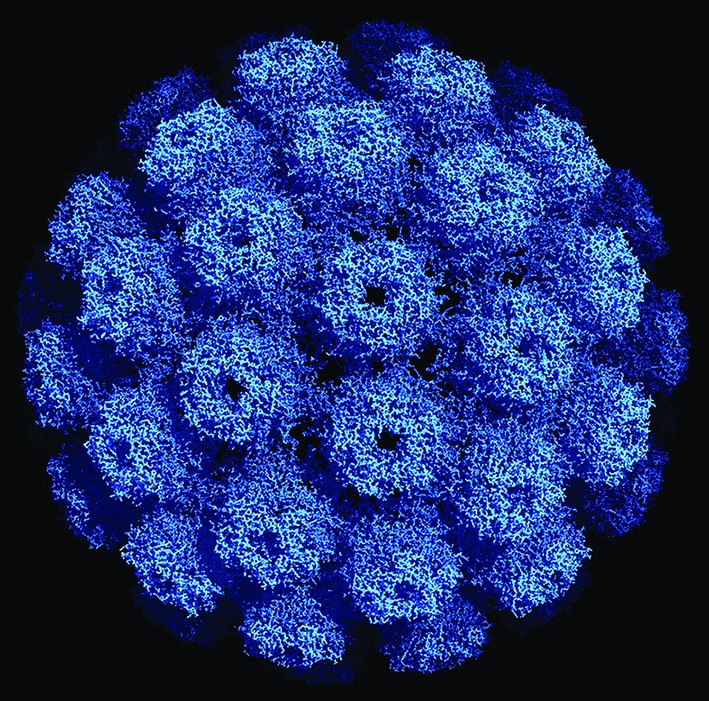
Three-dimensional structure of an SV40 particle at 3.1 ångstroms (Å) resolution obtained using X-ray diffraction (Protein Data Base ID 1SVA, [6]). The pentameric VP1 subunits are tied together by extended C-terminal arms. The diameter of this particle is about 500 Å or 50 nm. Reproduced with permission obtained from RCSB Protein Data Bank.

Virions are typically 40–45 nm in diameter and lack an envelope. The icosahedral capsid is constituted of 72 capsomers, each composed of five molecules of the major capsid protein VP1 (Table 1, Fig. 1). Minor capsid proteins are located at the internal face of the capsid [1].

**Table 1.** Characteristics of the family *Polyomaviridae*

Typical member:	simian virus 40 strain 776 (SV40-776) (J02400), species *Macaca mulatta polyomavirus 1,* genus *Betapolyomavirus*
Virion	Non-enveloped, 40–45 nm, icosahedral
Genome	Approximately 5 kbp circular dsDNA
Replication	Bidirectional from a unique origin of DNA replication
Translation	Early and late transcripts, alternative splicing, alternative ORFs
Host range	Mammals, birds and fish
Taxonomy	Four genera including more than 70 species

## Genome

**Fig. 2.**
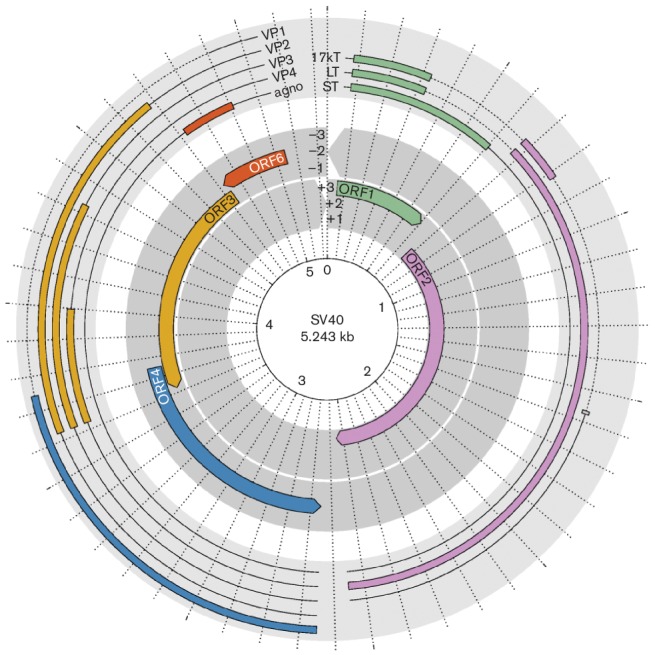
Genome organization and expression products of SV40. The inner layer depicts the six reading frames of the genome. Open reading frames (ORFs) are indicated by colours. The outer layer depicts spliced transcripts, with coding exons coloured according to the respective ORFs. Untranslated regions and introns are shown as solid and dashed lines, respectively. The radial axis is in units of 100 bp.

The circular, dsDNA genome of approximately 5 kbp is packed with cellular histones and divided into three functional domains: the early region encoding regulatory proteins, the late region encoding capsid proteins, and the non-coding control region, which contains the origin of DNA replication and the promoter/enhancer elements directing transcription of the viral genes ([1], Fig. 2).

## Replication

Well-studied mammalian polyomaviruses require cellular glycans as host cell receptors [1]. Viral decapsidation initiates in the cytosol and is completed in the nucleus, where replication and assembly occur. Viral transcription synthesizes a single precursor mRNA that is alternatively spliced to generate the major early proteins large T-antigen (LTAg) and small t-antigen (STAg), in addition to minor alternative proteins. LTAg of most polyomaviruses binds repeats of the 5′-GRGGC-3′ motifs, while LTAg of gammapolyomaviruses interacts with the palindromic motif 5′-CC(W)_6_GG-3′. LTAg possesses ATPase/helicase activity, and autoregulates transcription of the early genes. After replication has initiated, two major late transcripts are produced that are translated as the capsid proteins VP1 and VP2. Alternative start codon usage of the VP2 mRNA can produce VP3 [1]. Avian polyomaviruses possess a unique VP4 capsid protein [2], and some mammalian polyomaviruses produce an agnoprotein that is involved in transcription, virus maturation and egress. Mature virions are released by cell lysis or non-lytically. Polyomavirus infection can be symptomatic and may cause severe disease, including malignancy and organ failure, resulting in death.

## Taxonomy

### Alphapolyomavirus

This genus includes >30 species. Members infect humans and other mammals. Merkel cell polyomavirus and raccoon polyomavirus are so far the only members known to cause cancer in their natural host [3].

### Betapolyomavirus

This genus includes >20 species. Members infect mammals. The well-studied human polyomaviruses BK and JC are associated with nephropathy and progressive multifocal leukoencephalopathy, respectively [3].

### Gammapolyomavirus

This genus includes <10 species. Members infect birds. Some cause severe illness and even death, but oncogenicity has not been observed [3].

### Deltapolyomavirus

This genus includes the species *Human polyomavirus 6* and *Human polyomavirus 7*, members of which exhibit skin tropism, and *Human polyomavirus 10* and *Human polyomavirus 11*, members of which (MW polyomavirus and STL polyomavirus, respectively), are commonly detected in the gastrointestinal tract [3].

The general mode of polyomavirus diversification is co-speciation with their hosts. Recombination has shaped polyomavirus genomes and resulted in conflicting phylogenetic signals from the early and late genomic regions [4]. A distant evolutionary relationship of polyomaviruses to ssDNA viruses has been suggested on the basis of structural similarity in replicative proteins [5].

## Resources

Full ICTV Online (10th) Report: www.ictv.global/report/polyomaviridae.
